# Supplementary material for: Complexome profiling of the Chlamydomonas psb28 mutant reveals TEF5 as an early PSII assembly factor
Source: Plant Cell. 2025 Mar 17;37(6):koaf055. doi: 10.1093/plcell/koaf055 (PMC12164590; doi:10.1093/plcell/koaf055)
Supplement: koaf055_Supplementary_Data [file koaf055_supplementary_data.zip › psb28_interactive.zip!/plots/intensity/qualityPlots/peptides/FAP223_Cre17.g705000.t1.2.html]

### Cre17.g705000.t1.2

Trivial name: FAP223  
SeqCoverage [median]: 16.80  
Mass: 67.45 kDa  
  
 

| Sequence | MissedCleavage | Mass [Da] | wtIntensity | muIntensity | Score |
| --- | --- | --- | --- | --- | --- |
| DLKPENFLLSDR | 1 | 1445.75 | 687.23 | 3977.69 | 87.00 |
| DVALGSGK | 0 | 745.40 | 0.00 | 741.04 | 61.96 |
| ELFLEIDKDK | 1 | 1248.66 | 0.00 | 438.31 | 41.28 |
| FDENGDGVITR | 0 | 1221.56 | 0.00 | 305.33 | 86.90 |
| GTFGTTYSATNKK | 1 | 1374.68 | 518.83 | 0.00 | 38.19 |
| KGMQGLTDADVSR | 1 | 1376.67 | 1041.88 | 3275.25 | 103.76 |
| LVSAEEIGDVQR | 0 | 1314.68 | 1410.03 | 1848.62 | 141.95 |
| MKQFSAMNR | 1 | 1111.53 | 0.00 | 741.18 | 47.85 |
| TATAQTTNPYAPVSK | 0 | 1548.78 | 560.65 | 2258.88 | 149.23 |
|  |  |  | 4218.6 | 13586.3 |  |
